# Molecular Cloning of the Vitellogenin Gene and the Effects of Vitellogenin Protein Expression on the Physiology of *Harmonia axyridis* (Coleoptera: Coccinellidae)

**DOI:** 10.1038/s41598-017-14339-3

**Published:** 2017-10-24

**Authors:** T. Zhang, G. Zhang, F. Zeng, J. Mao, H. Liang, F. Liu

**Affiliations:** 1 Key Laboratory for Biology of Plant Diseases and Insect Pests, Institute of Plant Protection, Chinese Academy of Agricultural Sciences, Key Laboratory of Integrated Pest Management in Crops, Beijing, China; 2 School of Forestry, Northeast Forestry University, Harbin, Heilongjiang China

## Abstract

Vitellogenin (Vg), the main egg storage protein precursor, plays an integral role in many oviparous animals, including *Harmonia axyridis*, an important agent for the biological control of many insect pests. In this study, the full-length Vg gene of was cloned. The open reading frame (ORF) of *H. axyridis* Vg cDNA is 5,403 bp in length and encodes 1,800 amino acids, with a predicted molecular mass of 211.88 KDa (accession number in NCBI: KX442718). Recombinant protein (18 kDa) expressed by the cloned Vg gene was characterized, and the effects of the expression of this protein on the physiology of *H. axyridis* were investigated. We found that Vg fragment significantly increased the egg production of *H. axyridis*. Furthermore, we also found that the activities of trypsin and lipase in *H. axyridis* were significantly higher in the groups treated with Vg fragment compared with those of the controls. The data from this study also reveals that Vg expression has significant effects on the physiology of *H. axyridis* and leads to increased egg production in these insects. These results may have future implications for increasing the reproduction rates of beneficial insects.

## Introduction

Vitellogenin (Vg) is a critical precursor protein of egg yolk vitellin (Vn) that serves as an energy reserve in many oviparous species^1^. Vgs are primarily synthesized in fat cells in tissue-, sex- and stage-specific manners, secreted into the hemolymph and subsequently sequestered by competent oocytes via receptor-mediated endocytosis^2–4^. As such, Vg is an important element for the reproduction of oviparous animals and the proliferation of such populations.

Vg genes and cDNAs have been extensively reported in many animals, including both vertebrates^5–7^ and invertebrates (e.g., insects)^8–11^. Researchers have found that Vg is involved in oocyte maturation and development and is thus a critical factor for insect reproduction. Vgs have since been studied in many insects, including Lepidoptera, Diptera, Hymenoptera and Hemiptera^2,12–14^. However, the role of Vg has not yet been reported for any natural enemies of insect pests, including *Harmonia axyridis* (Coleopteran). *H. axyridis* is an important biological control predator for many insect pests^15^, such as aphids, mites, thrips, lepidopteran eggs and newly hatched larvae.

The food is ingested through the food canal and passed into the alimentary canal where it is further digested and absorbed by enzymes such as trypsin and lipase in insects^16–18^. Szenthe, B. *et al*. (2005) indicated that trypsin produced by pancreatic acinar cells as a trypsinogen, released into the intestine, and converted into active trypsin through the action of enterokinase or by autoactivation^19^. Trypsin is a hydrolysis protease found in digestive system of many insects, and trypsins and chymotrypsins are major endopeptidases in most insects^18,20^. Lipase not only plays an important role in the process of hydrolysis, absorption and metabolism of lipids and lipoproteins, but also relates to the growth and development of insects^20,21^.

The aims of this study were to clone the full-length Vg gene of *H. axyridis* and investigate its effects on *H. axyridis* reproduction (egg production and hatching) and biochemistry (general lipase and trypsin activities). Through a better understanding of the effects of Vg on the physiology of *H. axyridis*, we may develop improved techniques to increase the population of this important insect^22^.

## Results

### Molecular cloning and analysis of cDNA sequencing

The full-length *H. axyridis* Vg cDNA was cloned using RT-PCR. This gene contains a 5,403 bp ORF that encodes for 1,800 amino acids (accession number in NCBI: KX442718). According to the online SignalP 4.0 software used for sequence analysis, the Vg protein contains a signal peptide before amino acid 17 with a signal peptide cleavage site between amino acids 17 and 18. The theoretical pI is 4.71, and the molecular weight is 211.88 KDa.

A Vg-N domain in the Vg protein (amino acids sites: 38–753) was found using NCBI BLAST to analyze the amino acid sequence in the middle region of the Vg protein. A DUF1934 domain (amino acids sites: 793–1077) and a Willerbrand factor type D (VWD) domain were found in the C-terminus (amino acids sites: 1465–1651), and these are specific to the Vg structure^23^.

### Comparison of the *H. axyridis* Vg gene with the Vg genes of other insects

**Figure 1:**
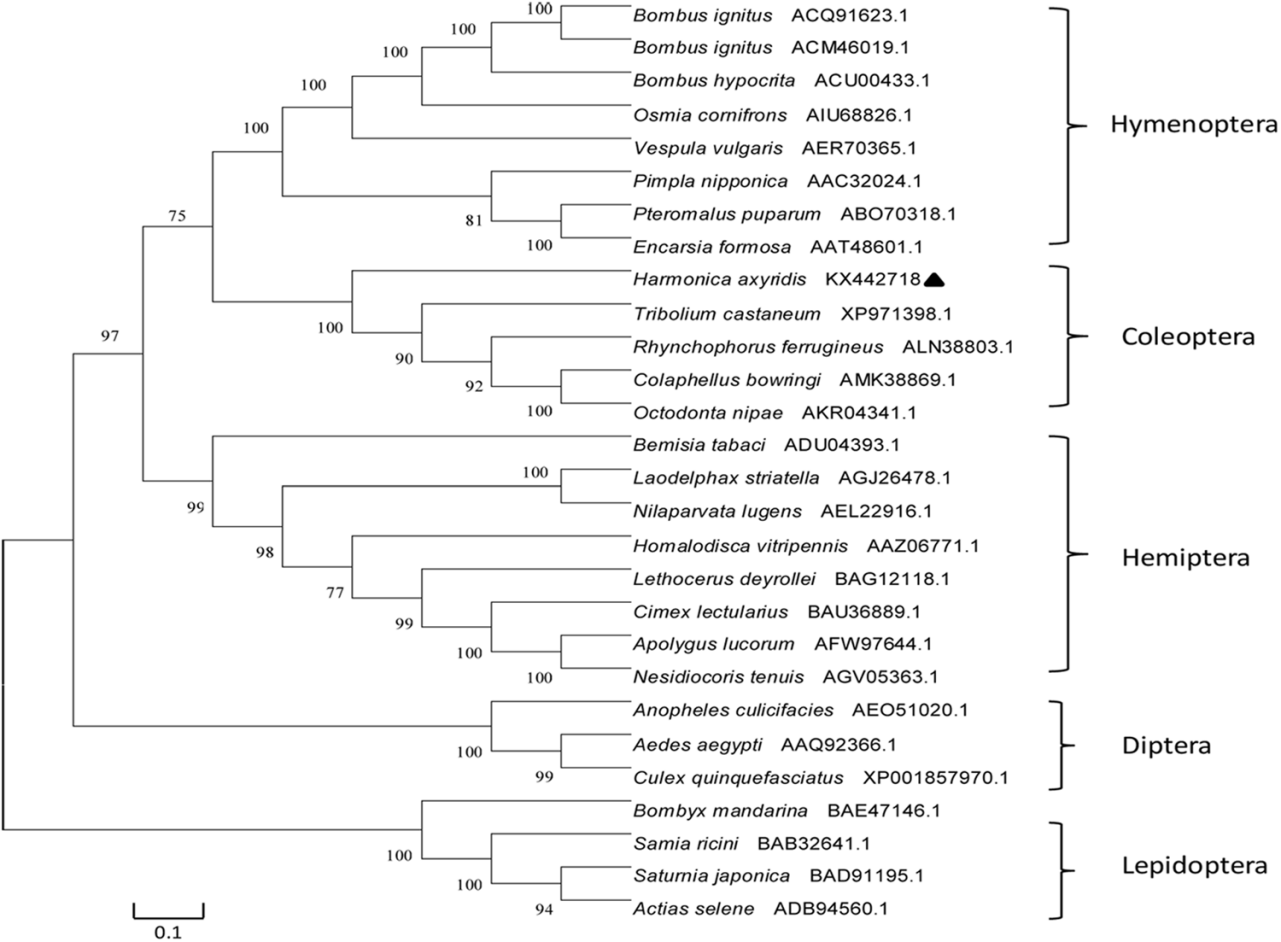
Homology tree of the *H. axyridis* Vg gene compared with other insects. Amino acid sequences from 26 insect species were used for phylogenetic analysis. A distance analysis and input for a neighbor-joining tree construction program are shown. BootstraP values (500 replications) are indicated at each node.

*H. axyridis* Vg was compared with the Vg of other insects using BLASTX. Through BLAST alignment and phylogenetic tree analysis, we found that the *H. axyridis* Vg gene and its amino acid sequence shares varying levels of homology with the following insects: *Tribolium castaneum* (38% homology)*, Rhynchophorus ferrugineus* (34% homology), *Bombus hypocrite* (28% homology) (Fig. 1). *H. axyridis* Vg is the most homologous with the Vg of *T. castaneum*.

### Expression and purification of Vg fragment

**Figure 2:**
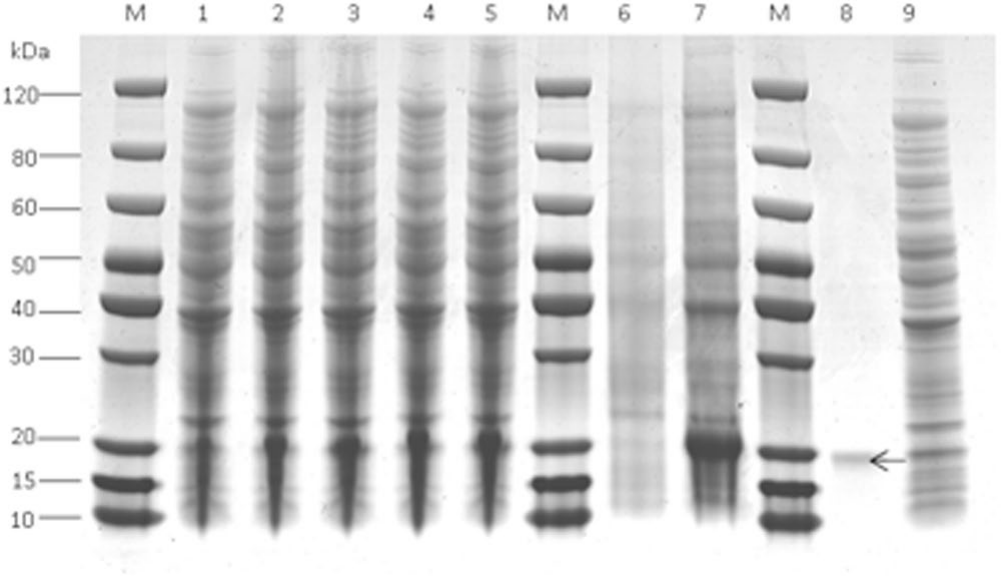
Expression and purification of the Vg fragment protein. Lanes 1–5: Different times after IPTG induction; Lane 6–7: Supernatant and sediment after sonication; Lane 8–9: Purified and non-purified Vg fragment protein.

The recombinant plasmid pET-28-VWD was transferred into *E. coli*, and its expression was induced with 0.5 M IPTG. The resultant recombinant protein was purified and examined on SDS-PAGE (Fig. 2). The level of protein expression increased with a longer induction time.

### The effects of the Vg fragment on *H. axyridis* reproduction

**Figure 3:**
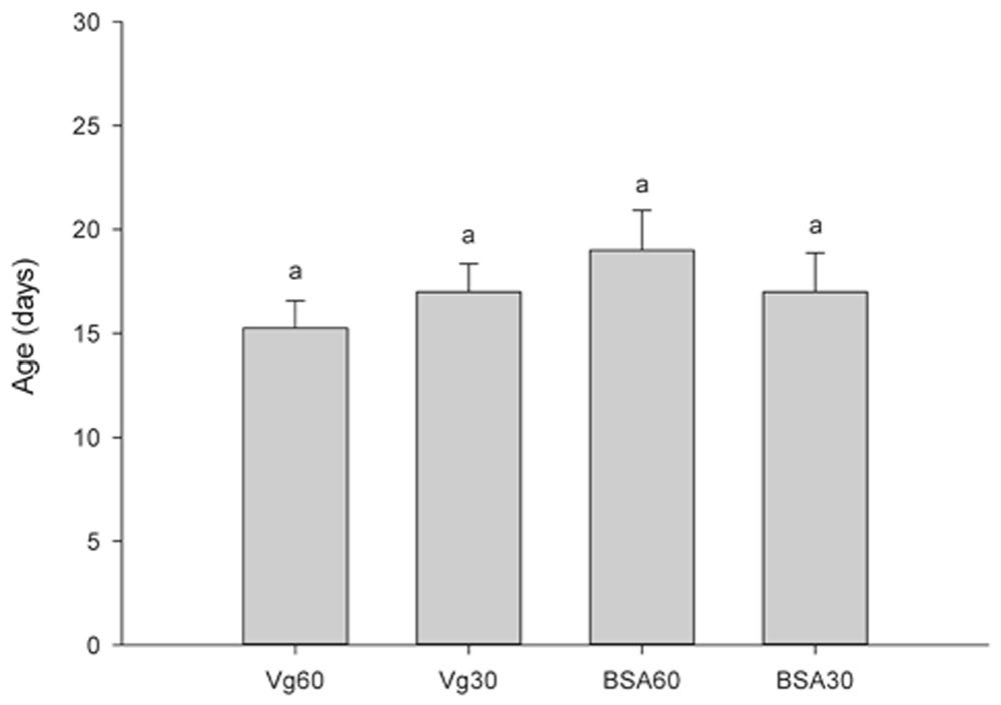
Pre-oviposition period of *H. axyridis*. Adults sustained on 60 μg/mL Vg, 30 μg/mL Vg, 60 μg/mL BSA and 30 μg/mL BSA. Mean ± SE is shown; bars with the same letter indicate that the values are not significantly different (*P* = 0.481).

**Figure 4:**
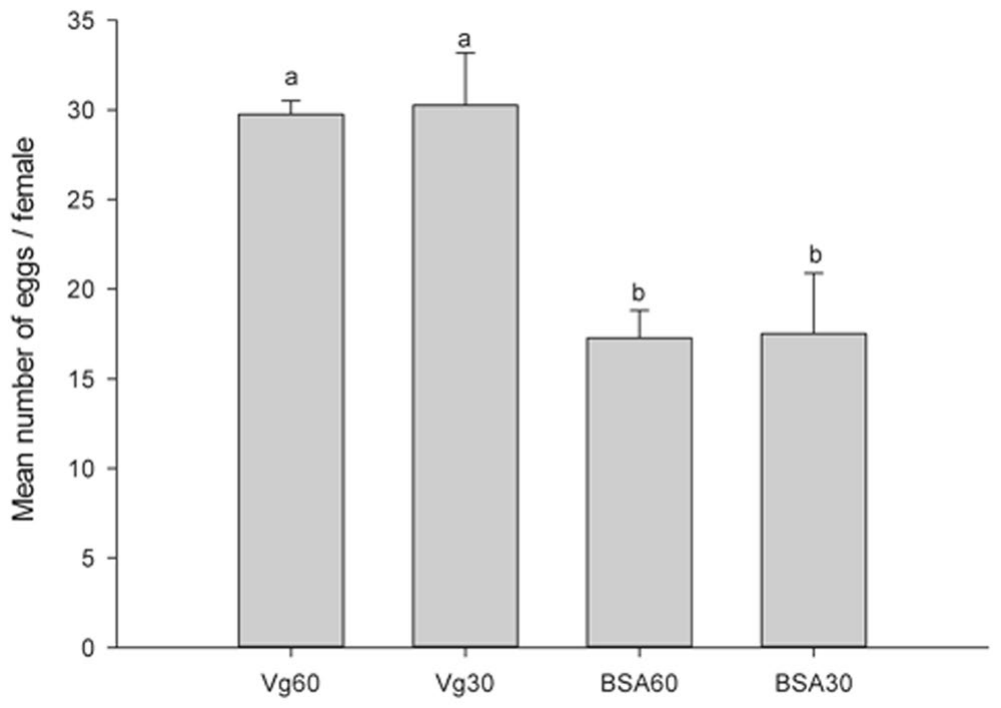
Total *H. axyridis* egg counts. Adults sustained on 60 μg/mL Vg, 30 μg/mL Vg, 60 μg/mL BSA and 30 μg/mL BSA (mean ± SE). Different letters above the bars indicate statistically significant differences (least significant difference test, *P* < 0.05).

**Figure 5:**
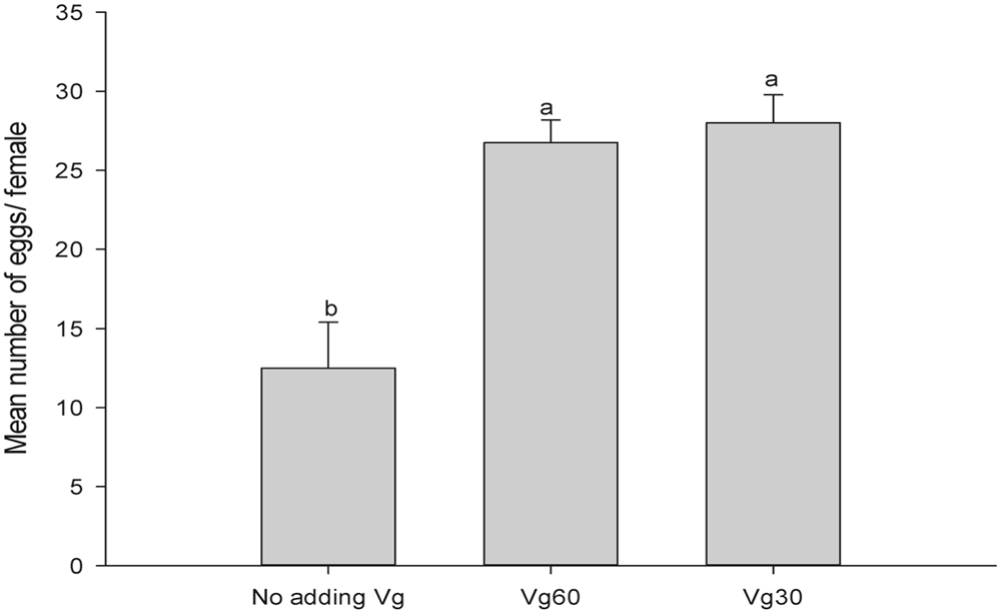
Total *H. axyridis* egg counts. Adults sustained on 60 μg/mL, 30 μg/mL Vg fragment protein and no adding Vg fragment protein (blank control). Mean ± SE is shown; bars with the same letter indicate that the values are significantly different (*P* = 0.044).

The effects of the 18 KDa recombinant Vg fragment on the development and reproduction of *H. axyridis* were evaluated. There were no significant effects on pre-oviposition time (the period from the emergency to the first egg-laying) (*F* = 0.876; *df* = 3, 15; *P* = 0.481) (Fig. 3) of *H. axyridis*. However, our results indicate that the total number of eggs in the treatment groups were significantly higher (*F* = 9.274; *df* = 3, 15; *P* < 0.05) than those in the control groups (Fig. 4). During the one month period, there were a total of 119 and 121 eggs produced from the groups treated with 60 μg/mL and 30 μg/mL of Vg fragment, respectively, and 69 and 70 eggs from the control groups treated with 60 μg/mL and 30 μg/mL of BSA, respectively. Similarly, the mean number eggs of 26.75 and 28 per female for treated groups were significant higher than that of the blank control (*F* = 16.301; *df* = 2, 11; *P* = 0.044) (Fig. 5). The groups treated with 60 μg/mL and 30 μg/mL of Vg fragment were 2.14- and 2.24–fold higher than that of the control (blank control and no adding Vg fragment). In addition, our data showed that the mean egg hatching rate (78%) in the treatment group (adding 30 μg/mL of Vg fragment) was higher than that (51%) of the control.

### Vg mRNA expression in *H. axyridis*

**Figure 6:**
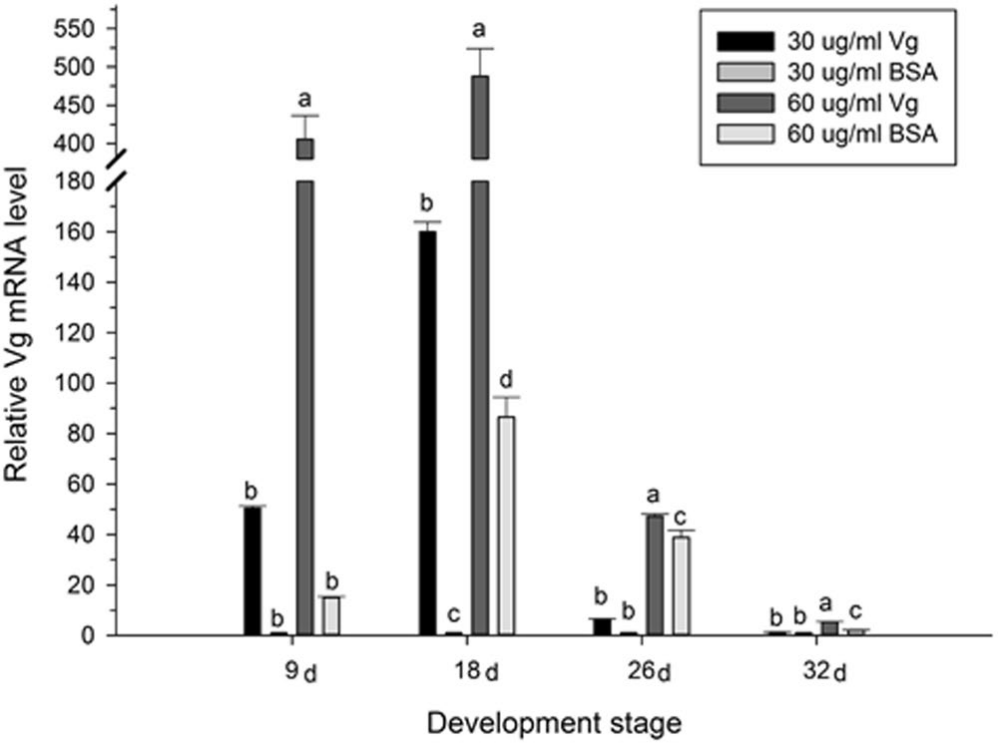
*H. axyridis* Vg gene expression. Adults sustained on 60 μg/mL Vg, 30 μg/mL Vg, 60 μg/mL BSA and 30 μg/mL BSA (mean ± SE). Different letters above the bars indicate highly significant differences (least significant difference test, *P* < 0.0001).

qRT-PCR was used to probe Vg gene expression during the different stages of the *H. axyridis* life cycle. The relative mRNA expression levels of the Vg gene showed markedly significant differences between the treatment groups and the control groups collected on the days 9 (*F* = 156.06*6; df* = 3, 11; *P* < 0.001), 18 (*F* = 131.693; *df* = 3, 11; *P* < 0.001), 26 (*F* = 257.967; *df* = 3, 11; *P* < 0.001) and 32 (*F* = 376.706; *df* = 3, 11; *P* < 0.001).The Vg mRNA expression levels in insects treated with 60 μg/mL Vg protein were 27-, 51-, 6-, and 2.5-fold higher than those of control groups on days 9, 18, 26, and 32, respectively. Similarly, the Vg mRNA expression levels in insects treated with 30 μg/mL Vg protein were 51-, 160-, 6-, and 1.3-fold higher than those of control groups on days 9, 18, 26, and 32, respectively (Fig. 6).

### Effects of the Vg fragment on lipase and trypsin activities

**Figure 7:**
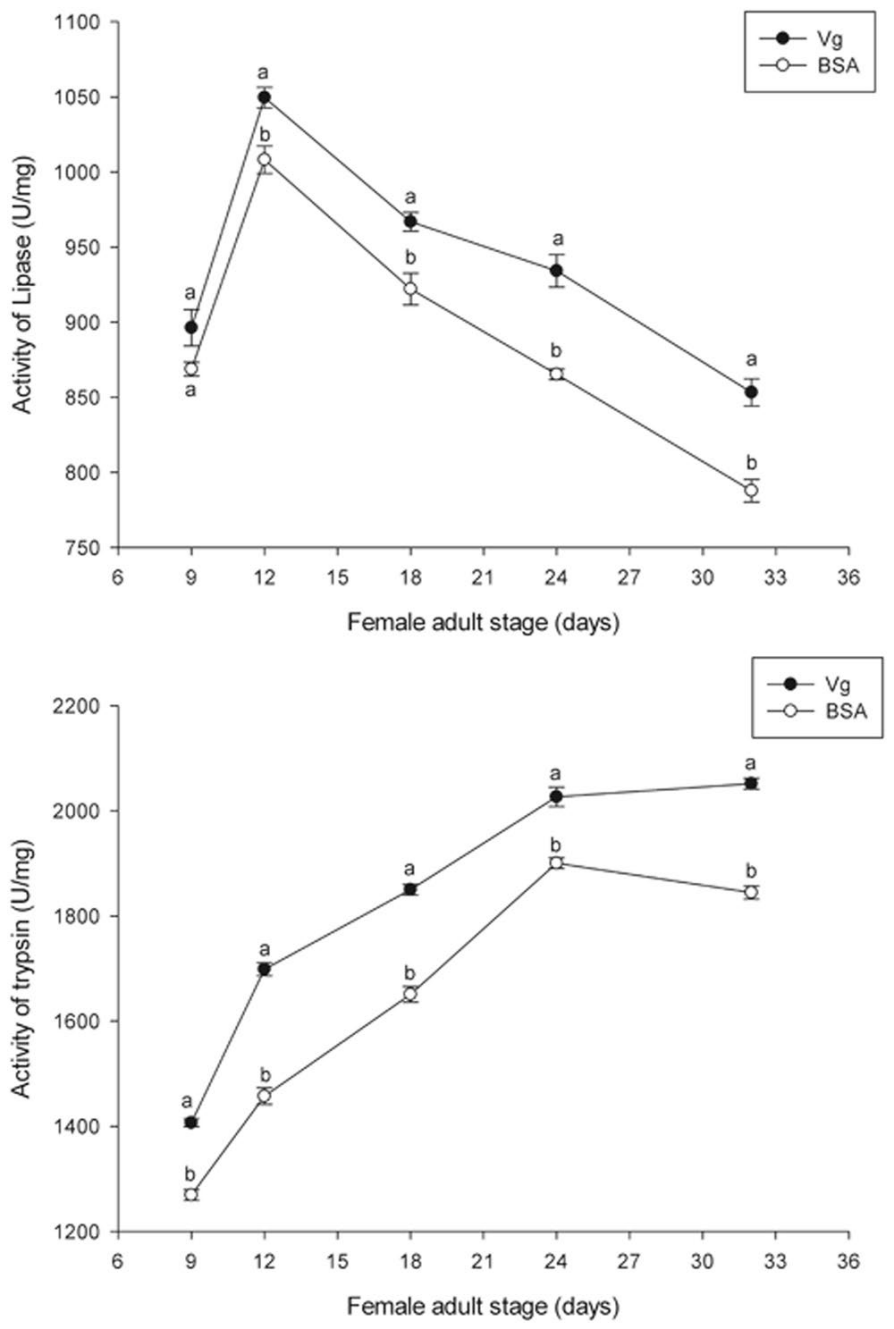
Lipase and trypsin activities in *H. axyridis*. Lipase and trypsin activities after treatment with 60 μg/mL Vg and 60 μg/mL BSA. General enzyme activity was analyzed by SPASS. Mean ± SE values for lipase and trypsin are significantly different (*P* < 0.05).

The effects of the Vg fragment on lipase and trypsin activities were determined. The lipase activities in the treatment group (adding 60 μg/mL Vg fragment) were significantly different from those of the control group on days 12 (*t* = 3.618, *P* < 0.05), 18 (*t* = 3.678, *P* < 0.05), 24 (*t* = 6.101, *P* < 0.05) and 32 (*t* = 5.543, *P* < 0.05), but there were no significant differences found on day 9. In addition, the trypsin activities in the treatment group were markedly different from those of the control group on days 9 (*t* = 10.957, *P* < 0.001), 12 (*t* = 12.162, *P* < 0.001), 18 (*t* = 11.088, *P* < 0.001), 24 (*t* = 6.013, *P* < 0.001), and 32 (*t* = 13.081, *P* < 0.001) (Fig. 7).

## Discussion

In recent years, the Vg gene of many insects has been cloned at different stages of growth and ovarian development^24^. Several studies have reported that the C-terminal and the VWD domain of Vg are related to the vitelline coat, which participates in fertilization as the binding partner of sperm proteases^25^. In this study, we cloned the complete cDNA of the *H. axyridis* Vg gene. Our primary structural analysis shows that the Vg-N, VWD, and DUF1943 domains are highly conserved in the Vg genes of oviparous animals^26,27^. Through our analysis using the NCBI Conserved Domain Search, we found three functional domains in the *H. axyridis* Vg gene. The VWD is a conserved domain and existed in the all known Vg protein sequences in insects. Liang *et al*. (2015) is also expressed VWD domain with Vg gene of *Geocoris pallidipennis* ^28^. The Vg gene plays an important role in embryonic development and serves as the main source of nutrients for oocyte growth and development^29^. Zeng *et al*. (1997) found that the expression of juvenile hormone was related to Vg production in insects^30^. This hormone has been reported to play an important role in the regulation of insect reproduction^31,32^. The regulation of Vg expression by hormones and social behavior is also found in honeybees^33,34^. However, the data from this study showed that the Vg gene expression can be affected by adding the VWD in the artificial diet to feed insects.

Niijima^35^ found that an artificial chemical diet sustains adult *H. axyridis* animals, however the diet did not assist in egg production. The major nutritional composition of artificial diet used in this study was analyzed and the soluble protein, sugar and lipid were about 10.9%, 1.95%, 1.72% respectively. There were no significant differences in total protein contents of artificial diets with different treatments (Supplementary information Fig. 1). Our results indicate that *H. axyridis* adults sustained on this artificial diet with the Vg fragment produced significantly more eggs than adults fed artificial diets with BSA. Furthermore, our addional experiment also show *H. axyridis* adults sustained on an artificial diet with the recombinant Vg fragment produced significantly more eggs than the control with no aditional protein. In other words, the recombinant Vg fragment promotes *H. axyridis* reproduction.

The Vg fragment, VWD, stimulates the egg production may due to the increase in egg related gene expression and addition of nutrients for egg development. Our hypothesis is that the Vg fragment stimulates the egg production may due to the increase in egg related gene expression in *H. axyridis* and additional nutrients for egg development. The qRT-PCR analysis of Vg gene expression levels during the different developmental stages of *H. axyridis* support this hypothesis. Our data show that Vg gene expression is increased upon treatment with the feeding of VWD. Vg gene expression in female adults increased by day 9 (Fig. 6 and Supplementary information Fig. 2) after emergence, and it reached a maximum level on day 18, at which point it decreased gradually until day 32 after emergence. This trend is consistent with egg production, and the number of eggs laid by *H. axyridis* female adults was higher for animals in the treatment groups compared with those of the control groups. The Vg protein can be detected 1 day after Vg mRNA expression begins^36–40^ and is a main nutritional source stored in ovary. As such, high Vg gene expression promoted by the VWD leads to increased protein levels of Vg in the ovaries. Therefore to promote the Vg expression will help egg production. Moreover, we have calculated and compared the percentage of each of the 20 amino-acids in Vg, the VWD fragment and BSA (Supplementary information Table 1). The results indicate that the percentage of amino-acids, Gly, Thr, Tyr and Val were higher in VWD fragment were much higher compared with those of BSA. This may be the reason of the increase of egg production in the VWD treatment, the treatment may provide more nutrients than the BSA treatment for egg development and production.

**Table 1:** Primers used in RT-PCR and qPCR.

Gene	Forward (5′-3′)	Reverse (5′-3′)
HaVg1	AACTGGGAGGYCAAYATSVTC	GTDACDGAVCTTTGAAYGRTG
HaVg2	AAATDCCCAGMACHCAAGGHA	GCTGAAGTCDGGRYTBVCHCC
HaVg3	TTATGGATCGTGTAGCAGGAAAA	GACCACGGACTCTGTTGCGCA
HaVg-F	TTACAGCCAAGCCTACCACA	
HaVg-R		TGGGTGGATGTCGTAGGTG
PmVg	TTACAGCCAAGCCTACCACA	AGAGGTTGCGGATGTCAGAA
18S	GGATTCGAAGCGCTTGGATT	CGCAGACAATCCCGAAAGAG

In addition, the amount of egg production is closely related to the nutrients for egg development. Many researchers have showed that Vg is one of important source of nutrition for embryo development (Tufail and Takeda)^24^, and Vg level is related to the growth and development of oocytes and egg production (Zeng *et al*., 1997^41^; 2000^29^). Like all oviparous animals, insects provision their eggs with proteins, lipids, carbohydrates, and other resources for the sustenance of the developing embryo (Sappington *et al*.^42^). The vitelloenin promotes the transport of carbohydrates, lipids and hormones, these nutrients can play a certain role in insect vitellogenin^37^. The treatment (feeding of VWD) increases the Vg gene expression, produces more Vg in *H. axyridis* female adults and results in an increase in more nutrients for egg development, so feeding of a Vg fragment can stimulate oogenesis and egg production. This may explain that the female feeding of VWD laid more eggs than the control. The results of the study suggest that the protein concentration of Vg in female adults might be used as a molecular marker to predict the fecundity of *H. axyridis*.

Furthermore, the results of this study also reveal that trypsin and lipase activities in *H. axyridis* were significantly higher in the treatment groups compared with those of the control groups (Fig. 7 and Supplementary information Fig. 3). This is the same as those reported by other researchers that food quality affects insect biochemistry^43^. Zeng and Cohen^44^ found that certain food can induce enzyme activity. The different component of artificial diets may result in differed activity of digestive enzymes; however, the most important reason for this may be that the Vg fragment stimulates the nutritional related gene expression and then simulates more trypsin and lipase activities.

In summary, the results from current study show that Vg fragment in artificial diets significantly alters the physiology of *H. axyridis* and increases egg production in the experimental insects. These results suggest that the Vg fragment increases the insects’ food quality. The techniques developed in this study have potential applications for increasing the population of beneficial insects.

## Methods

### Insects and Sample Preparation

*H. axyridis* insects were raised in a growth chamber (RXZ, Ningbo, China) under controlled conditions at 25 ± 1 °C with a photoperiod of 16 L: 8D and 70 ± 5% RH in a climatic incubator throughout all developmental stages. Total RNA was extracted from *H. axyridis* adult females using Tranzol reagent, according to the manufacturer’s instructions. Genomic DNA was removed by DNase I (Transgene, Beijing, China). RNA integrity was determined by agarose gel electrophoresis, which showed clear bands of 18S and 28S.

### Cloning, expression and purification of growth-promoting Vg fragment

First, the full-length cDNA of vitellogenin (Vg) was cloned. The double-stranded cDNA was synthesized from 4 μl of total RNA using the cDNA Synthesis Super Mix Kit (TransGen Biotech, Beijing, China) and oligo (dT) 18 primer according to the manufacturer’s instructions. The degenerate primers were designed according to the conserved domain structure of insects. Briefly, the primers are designated as HaVg1 and HaVg2 (Table 1). The PCR amplification was performed with high-fidelity Taq enzyme (TransGen Biotech, Beijing, China) under the following amplification conditions: denaturing at 94 °C for 3 min, followed by 35 cycles at 94 °C for 30 s, 52 °C for 30 s, and 72 °C for 1 min, with a final 10 min extension for the last cycle. The amplified fragments were cloned into a pMD^TM^ 19-T vector (Takara, Dalian, China) and transformed into competent DH5α cells (TransGen Biotech, Beijing, China). Positive colonies were sequenced using T7 primers.

The full-length Vg cDNA was obtained by Rapid Amplification of cDNA end (RACE) methods using the SMART^™^ RACE cDNA Amplification Kit (Takara, Dalian, China) according to the manufacturer’s instructions. Gene-specific primers, HaVg-F (for 3′RACE) and HaVg-R (for 5′RACE) were designed corresponding to the sequence of the known Vg gene of *H. axyridis* (Table 1). The 5′ and 3′ end amplifications were carried out with the Advantage 2 Polymerase mix (Clontech, USA). The PCR conditions were as follows: 94 °C for 3 min, followed by 30 cycles of 94 °C for 30 s, 65 °C for 30 s and 72 °C for 1 min, with a final 10 min extension for the last cycle. The amplified PCR products were analyzed by 1% agarose gel electrophoresis and cloned into a pMD^TM^ 19-T vector (Takara, Dalian, China) for sequencing. The overlapping sequences of the above PCR fragments were assembled to obtain the full-length sequences.

The sequence of the cloned Vg gene was subjected to a homology search using the NCBI’s Basic Local Alignment Search Tool database: http://www.ncbi.nlm.nih.gov/. The signal peptide was predicted using the SignalP 4.0 Server: http://www.cbs.dtu.dk/services/SignalP/. Protein molecular weight and isoelectric point were calculated using the ExPASy: http://www.expasy.org/tools/pi_tool.html. The conserved domain structure was predicted by the Conserved Domain Database (CCD) search: http://www.ncbi.nlm.nih.gov/Structure/cdd/cdd.shtml.

Third, the VWD domain was expressed and collected. The specific primers of HaVg3 were designed based on the known sequence of Vg, with the first strand of *H. axyridis* cDNA used as a template for PCR amplification. The amplification resulted in a PCR product containing a 543-bp fragment, which was then cloned into a pEASY-T1 vector (TransGen Biotech, Beijing, China) to produce the pEASY-Vg plasmid. The pEASY-Vg and pET-28 plasmids were double digested with *BamH*I and *Nhe*I restriction endonucleases, respectively. After linking the double-digested pEASY-Vg and pET-28a, the recombinant expression plasmid pET28a-HaVg was sequenced. The pET28a-HaVg plasmid was then transformed into *E. coli* BL21 cells (TransGen Biotech, Beijing, China), which were grown at 37 °C in 1,000 mL of LB medium with 100 μg/mL kanamycin until an OD_600_ value of 0.6–0.8 was reached. After induction with 0.2, 0.4, 0.6 and 0.8 mM of isopropyl-β-D-thiogalactopyranoside (IPTG) for 6 h at 37 °C, the BL21 cells were harvested by centrifugation at 6,000 × g for 20 min at 4 °C. The cell pellet was resuspended in 30 mL of 0.01 M PBS (136.89 mM NaCl, 2.68 mM KCl, 4.02 mM Na_2_HPO_4_, 1.76 mM KH_2_PO_4_, pH 7.4) and sonicated at 4 °C for 20 min using cycles of 3 s on and 5 s off with 40% amplitude.

The inclusion bodies were future re-suspended in 20 mL buffer B (50 mM Tris-HCl, 100 mM NaCl, 0.5 mM EDTA, 2 M urea and 1% Triton X-100, pH 8.0), room temperature in 0.5 hours and then centifuged at 12,000 × g at 4 °C for 30 min. The pellets were washed twice with buffer C (100 mM Tris-HCl, 500 mM NaCl, 6 M urea, pH 7.4) with 10 mM imidazole, and keep in 4 °C for 24 h and centifuged at 12,000 × g at 4 °C for 30 min. The supernatant was filtered through 0.22 μm filter membrane, and used a Ni-NTA-Sepharose Column (GE Healthcare). The column was washed with buffer D (buffer C with 250 mM imidazole). Finally, The purified proteins were dialyzed at 4 °C by dialysis buffers in different gradient^45^. The concentration of the purified Vg fragment was determined using a BCA protein assay kit (Pierce, Rockford, IL); BSA was used as the standard, and the concentration of the target protein sample was calculated against the standard curve.

### Effects of Vg fragment on the reproduction

The purified soluble Vg fragment was included as part of an artificial diet, which was fed at different concentrations (30 μg/mL and 60 μg/mL) to adult *H. axyridis* animals. The diets of control groups were supplemented with equivalent concentrations of BSA proteins (Jiangchen, Beijing, China). Male and female adults were paired within 1 day after eclosion and placed in a Petri-dish (Jiangchen, Beijing, China), and the artificial diet was replaced every day with fresh food. At least 15 pairs of adults were used for each experiment. The total amount of eggs produced within a one-month period by each pair of adults in the treatment or control groups was recorded, and the egg hatching rates were also determined. Animals were randomized to 4 different experimental groups, and 4 replications were done for each group.

### Vg mRNA expression in female adults

The expression level of PmVg and 18S transcripts at different development stages is shown Table 1. Total RNA was isolated from female adults and treated with RNase-free DNase I at 37 °C for 30 min using the DNase I kit (Takara, Dalian, China). A reaction volume of 20 μL was used: 0.5 μL forward and reverse primers, 1 μL cDNA, 8 μL nuclease-free water, and 10 μL 2X iTaq universal SYBR Green supermix (BIO-RAD, California, American). This real-time PCR reaction produced 200 ng total RNA, which was analyzed by a 7500 real-time system (Applied Biosystems, California, USA). The qPCR reaction conditions were as follows: 95 °C for 2 min, 40 cycles of 95 °C for 15 s and 60 °C for 1 min. The housekeeping gene 18 S was used for comparison in the 2^−ΔΔCt^ qPCR method. Means and standard errors for each time point were obtained from the average of four independent samples. Individual animals were randomized into 2 treatment groups, and 3 replications were performed for each treatment.

### Effects of the Vg fragment on lipase and trypsin activities

Trypsin activity was determined according to the method described by Erlanger *et al*.^46^. BAPNA and BTPNA were used as chromogenic substrates. A total of 20 μL of extract was mixed with 150 μL chromogenic substrate and incubated at 37 °C for 45 min. Crude extract samples (20 μL) and substrate were mixed using a spectrophotometer (Flexstation 3, California, USA), and the absorbance was read at 410 nm. Trypsin activity was recorded as absorbance units per U/mg of protein, and the measurements were repeated three times for each sample. Individual animals were randomized into 2 treatment groups, and 3 replications were performed for each treatment. This same method was used for measuring lipase activity, but nitro phenyl palmitate (p-NPP) served as the chromogenic substrate.

## Electronic supplementary material

Supplementary information
