# Supplementary material for: Molecular Cloning of the Vitellogenin Gene and the Effects of Vitellogenin Protein Expression on the Physiology of Harmonia axyridis (Coleoptera: Coccinellidae)
Source: Sci Rep. 2017 Oct 24;7:13926. doi: 10.1038/s41598-017-14339-3 (PMC5654974; doi:10.1038/s41598-017-14339-3)

Molecular Cloning of the Vitellogenin Gene and the Effects of Vitellogenin Protein Expression on the Physiology of *Harmonia axyridis* (Coleoptera: Coccinellidae)

T Zhang[1,2], G Zhang[2], F Zeng[1*], J Mao[1], H Liang[1], F Liu[1]

Supplementary information Tab 1. Each of the 20 amino-acids percentages in the VWD fragement, BSA and full-length Vg.

	VWD(%)	BSA(%)	Vg(%)
Ala(A)	2.2099	7.743	4.7222
Arg(R)	4.4199	4.2834	5
Asn(N)	3.8674	2.3064	4.8889
Asp(D)	6.6298	6.5898	4.8333
Cys(C)	1.105	5.7661	0.8333
Gln(Q)	3.8674	3.2949	5.5556
Glu(E)	6.0773	9.7199	6.9444
Gly(G)	6.0773	2.8007	5
His(H)	0.5525	2.8007	2.4444
Ile(I)	4.4199	2.4712	5.5556
Leu(L)	6.6298	10.7084	7.4444
Lys(K)	8.2873	9.8847	7.7222
Met(M)	2.2099	0.8237	2.2222
Phe(F)	2.2099	4.9423	3.6111
Pro(P)	4.4199	4.6129	3.7778
Ser(S)	9.9448	5.2718	9.2222
Thr(T)	7.1823	5.7661	5.6667
Trp(W)	0.5525	0.4942	0.6667
Tyr(Y)	7.1823	3.4596	5.5
Val(V)	12.1547	6.2603	8.7778

Supplementary information Fig 1. Total protein contents of artificial diets with different treatments (60 μg/mL) (*F* = 0.065; *df* = 2, 8; *P*=0.937, ANOVA).

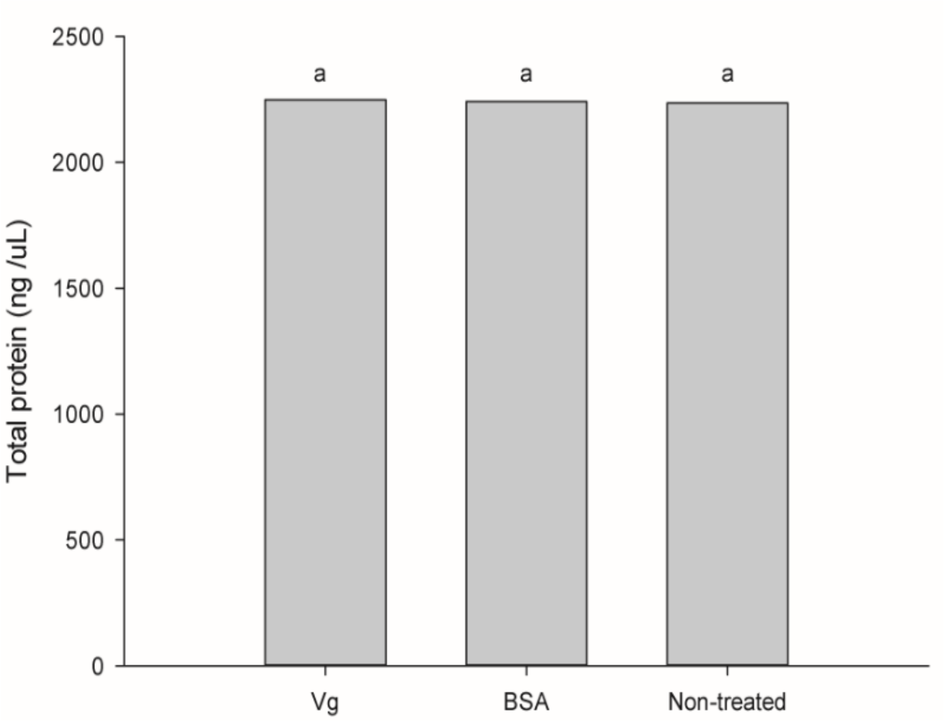

Supplementary information Fig 2. Vg gene expressions with different treatments (60 µg/mL) (*F* = 6417.106; *df* = 2, 8; *P* < 0.0001, ANOVA).

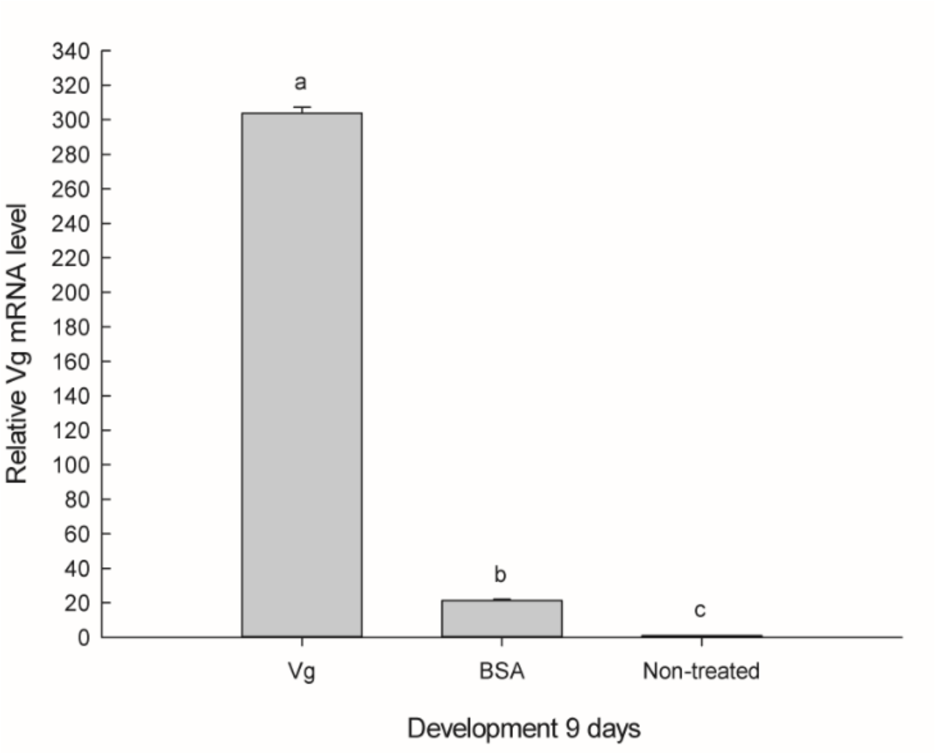

Supplementary information Fig 3. Lipase (*F* = 20.968; *df* = 2, 8; *P* = 0.002, ANOVA) and Trypsin (*F* = 189.763; *df* = 2, 8; *P* < 0.0001, ANOVA) activities with different treatments (60 μg/mL).

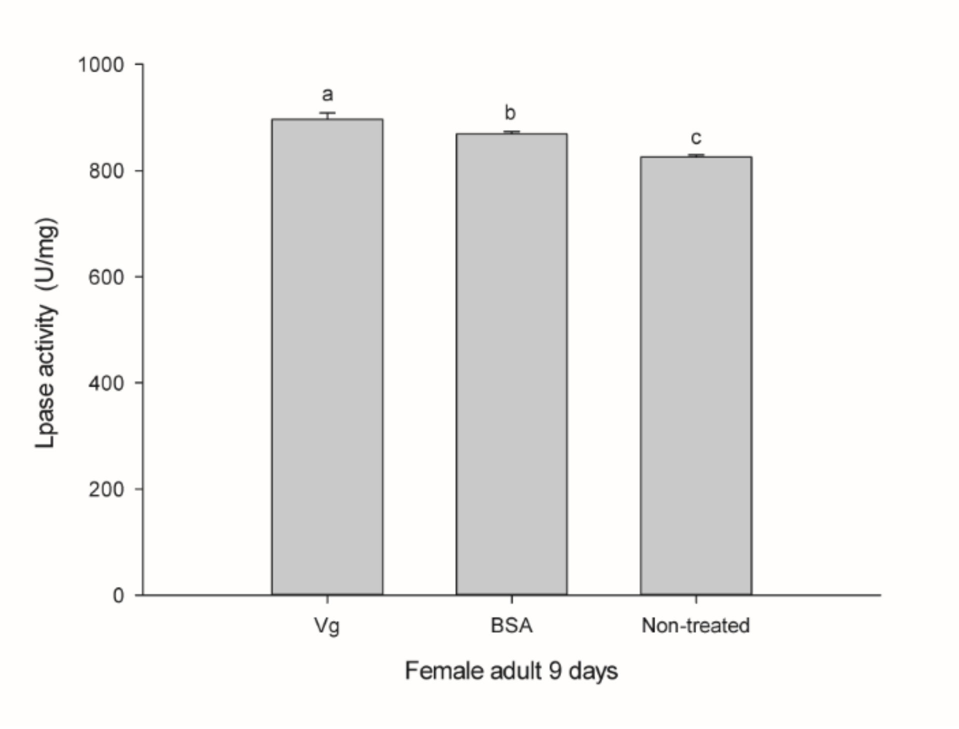

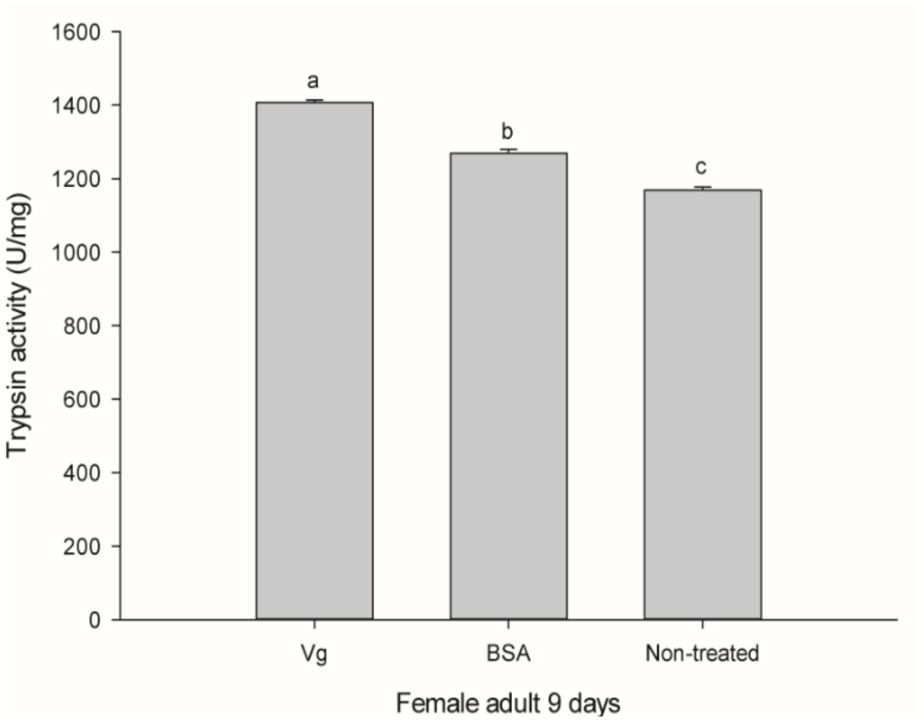

Supplement: Supplementary file 1 — Supplementary information [file 41598_2017_14339_MOESM1_ESM.pdf]
